# Rare within Rare: A Girl with Severe Haemophilia A and Turner Syndrome

**DOI:** 10.3390/jcm12237437

**Published:** 2023-11-30

**Authors:** Cristina Blag, Margit Serban, Cristina Emilia Ursu, Cristina Popa, Adina Traila, Cristian Jinca, Ciprian Tomuleasa, Madalina Bota, Ioana Ionita, Teodora Smaranda Arghirescu

**Affiliations:** 1 Pediatric Discipline, Department of Mother and Child, Iuliu Hațieganu University of Medicine and Pharmacy, 400177 Cluj-Napoca, Romania; cristinablag@yahoo.com (C.B.); bota.madalina@gmail.com (M.B.); 2 Onco-Hematology Research Unit, Romanian Academy of Medical Sciences, Children Emergency Hospital “Louis Turcanu” Timisoara, European Hemophilia Treatment Centre, 300011 Timisoara, Romania; mserban@spitalcopiitm.ro (M.S.); dr.popacristina@gmail.com (C.P.); 3 Discipline of Genetics, “Victor Babes” University of Medicine and Pharmacy Timisoara, 300041 Timisoara, Romania; 4 Medical Centre for Evaluation Therapy, Medical Education and Rehabilitation of Children and Young Adults, European Hemophilia Treatment Centre, 305100 Buzias, Romania; adinatraila@yahoo.com; 5 Department of Pediatrics, Division of Onco-Hematology, “Victor Babes” University of Medicine and Pharmacy Timisoara, 300041 Timisoara, Romania; cristian_jinca@yahoo.com (C.J.); sarghirescu@yahoo.com (T.S.A.); 6 Department of Hematology, Research Center for Functional Genomics and Translational Medicine, Iuliu Hatieganu University of Medicine and Pharmacy, 400012 Cluj Napoca, Romania; ciprian.tomuleasa@gmail.com; 7 Department of Hematology, “Victor Babes” University of Medicine and Pharmacy Timisoara, 300041 Timisoara, Romania; ionita.ioana@umft.ro

**Keywords:** severe haemophilia A in female, Turner syndrome, Isochromosome Xq

## Abstract

A coincidental occurrence of severe haemophilia A and Turner syndrome in a female person is extremely rare (less than 10 cases published). In such challenging cases, a multidisciplinary approach based on medicine of precision with full access to genetic and bio-molecular exploration is indispensable. The article presents an eight-year-old girl, with a family history of haemophilia, without significant disease signs (only post-dental extraction bleeding and a shorter stature). Discordantly, however, the investigations revealed a challenging condition: a genotype of 46,X,i(Xq), with an Isochromosome Xq responsible for the Turner syndrome and simultaneously, for the detrimental transformation, interfering with X chromosome inactivation, of an obligate hemophilia carrier into a severe hemophilia case—two distinct and provocative diseases.

## 1. Introduction

Haemophilia is an X-recessive congenital hereditary coagulopathy affecting almost only men. Due to Factor (F) VIII or IX deficiency, it has a high predisposition to uncontrolled spontaneous or posttraumatic bleedings. Bleeding in females is a relatively common event in a lifelong experience. Its frequency is estimated in minors at 5.3–7.7 and majors at 0.06 events per 100 persons-years, respectively, increasing with age-related comorbidities [1,2]. The landscape of congenital hereditary causes is likely to be large, including a long list of autosomal dominant inherited defects, but rarely, also recessive X-linked ones. Haemophilia belongs to this group [3,4,5]. Historically, it has been considered as an exclusively male condition, and only since the 20th century has it been accepted that females may be symptomatic carriers of haemophilia. The progress registered in haemostatic laboratory exploration, along with the development of the genetic investigations, has led to the discovery of over 3000 unique mutations in the factor VIII gene (F8) and more than 2000 in the factor IX gene (F9). The integration of these findings into clinical practice has enlarged the number of reported females with haemophilia. This progress has paved the way for precision medicine, revealing the genetic causes and, based on convincing arguments, defining the various phenotypes of female hemophilia and their potential evolution [4,5,6,7]. In addition to the pathologic X inherited from the father, haemophilic females have another pathologic X chromosome from their mother. When one of the parents is healthy and missing a pathologic X chromosome, like our patient, an additional spontaneous mutation or non-functioning X chromosome, like isochromosome Xq, is most probably.

In the following section, we would like to present such a patient, illustrating the complexity and heterogeneity of his haemophilia condition. Informed consent and ethics committee approval have been obtained.

## 2. Case Presentation

An 8-years- and 6-month-old girl visited the clinic after abnormal hemostatic parameters (activated partial thromboplastin time (aPTT) of 52 s) were discovered during ambulatory investigations prompted by prolonged post-dental extraction bleeding. The physical examination showed nothing remarkable being without any dysmorphic features. She had a height of 118 cm (percentile 5, N-126.7 ± 5.8 cm), a weight of 20 kg (N = 25.8 ± 4.3 kg), and a BMI of 14.36 kg/m^2^ (percentile 15). Her pulse rate was 80/min, blood pressure 90/40 mmHg, respiratory frequency 20/min and SpO-98%. The EKG was normal, and she had mild myopia in both eyes (−0.75). Except for the recent dental bleeding, her medical history showed no notable events, and there were no occurrences of muscular, soft tissue, or joint bleeding. But her family history revealed in her father and his two brothers a moderate form of haemophilia A, both at the time of writing undergoing on-demand replacement therapy with factor concentrate. In this situation, our patient was considered an obligate carrier, and extended haemostatic exploration was initiated. The laboratory tests revealed the following: aPTT—47.5 s (N = 24–32 s), prothrombin time—13.3 s (N = 11–20 s), thrombin time—13.1 s (N = 9–14 s), FVIII repeatedly <1% (N = 70–150%), FIX—57.4% (N = 50–120%), factor von Willebrand activity—48.4% (N = 50–150%), von Willebrand antigen—46.5% (N = 50–150%), ristocetin cofactor—75% (N = 50–140%); the blood count and renal and hepatic examinations were normal. Considering the range of her FVIII level, we had to change the diagnosis to severe Haemophilia A.

Bio-molecular examinations assessed the substrate of the disease. Whole exome sequencing (WES), one of the most extensive genetic tests, was performed. As primary findings, it revealed a double heterozygote for a pathogenic variant and another likely pathogenic variant, encoding coagulation factor genes, F8 and F13, respectively. The first pathogenic missense mutation of F8 was detected within the last base of exon 14, at codon 391—c1172G>A p.R.391H heterozygous (replacing Arginine with Histidine) in our patient and concurrently in her father. This mutation was already assessed in several individuals affected by haemophilia A, but is absent in the general population. The second likely pathogenic variant F13A1, a splicing mutation, was also detected as a primary finding. This variant has been reported in homozygous and compound heterozygous individuals with severe congenital factor XIII deficiency. Still, the FXIII activity in our patient was in the normal range (66.7–70%).

Another molecular change as a secondary finding, a copy number loss (CNV), encompassing the cytoband p11.23-q12, was also registered in our patient. This anomaly can suggest a non-random X inactivation leading to skewed X inactivation. Secondary skewing occurs when an X-linked mutation affects cell proliferation or survival. It has an impact on X-linked diseases having as a medical significance the amplification of the disorder connected to the X chromosome, like haemophilia, leading to a more severe clinical or biological expression.

**Figure 1:**
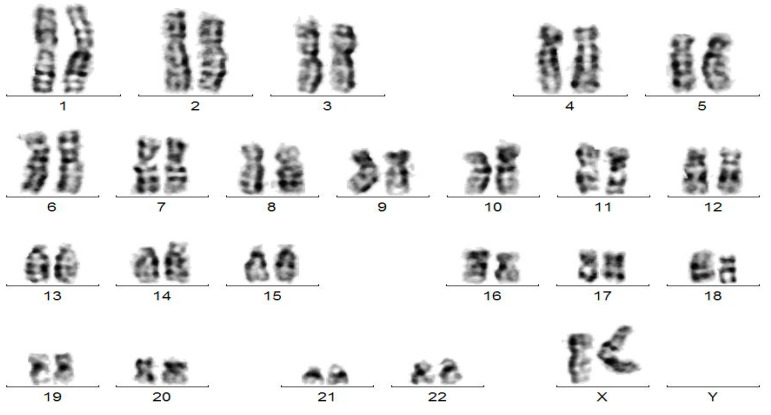
G-banding karyotype of the patient with Isochromosome X-46,X,i(Xq).

**Figure 2:**
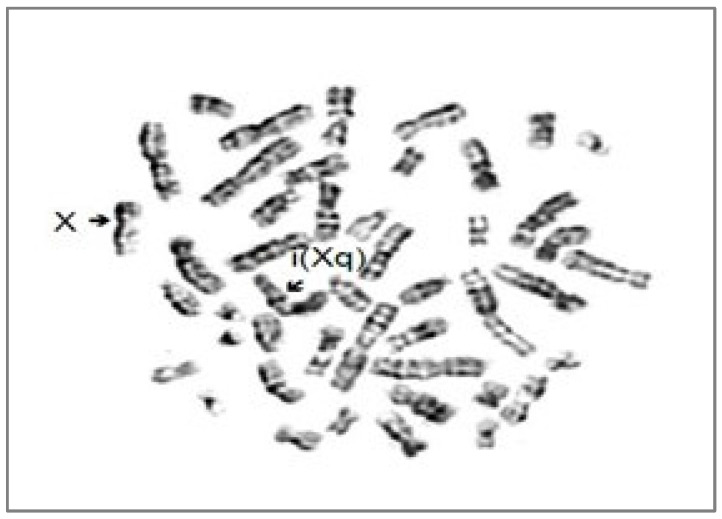
G-banding metaphases of the patient with Isochromosome X-46,X,i(Xq).

In addition to these biomolecular explorations, a cytogenetic investigation was carried out. A monosomy Xp and a trisomy Xq, with one short arm and three long arms in every cell were detected (Figure 1 and Figure 2). Considering this cytogenetic result karyotype 46,X,i(Xq), the patient was additionally diagnosed with Turner syndrome with a structure X chromosomal aberration, an Isochromosome X.

Finally, based on a comprehensive review of our exploratory results, a diagnosis of severe haemophilia A associated with Turner syndrome (Table 1) was concluded. Our patient initially was considered as an obligate carrier for haemophilia A (father with a moderate form of the disease), and was diagnosed with severe form of haemophilia A, due to her FVIII level (˂1%), her biomolecular finding (missense mutation on exon 14, codon 391), and an associated chromosomal abnormality (46,X,i(Xq). This can be responsible for a skewed X inactivation (XCI) and for the change from a moderate to a severe form of haemophilia A. The structural anomaly of Isochromosome X is also a substrate of Turner syndrome. Replacement prophylaxis with EHL (extended half-life) factor VIII has been started and the patient was confronted only with minor posttraumatic bleedings. What concerns her Turner syndrome, besides the short stature (corresponding to percentile 5 for her age), she remained without other phenotypic, neurocognitive, organic, metabolic, or autoimmune alterations.

## 3. Discussion

Historically, haemophilia has been considered for centuries as a rare inherited coagulation disorder, affecting exclusively male subjects. Both statements are now subjects of controversy. The progress registered in the haemostatic laboratory and in the field of bio-molecular exploration has changed the horizon of epidemiological data, increasing the number of persons with haemophilia (PwH), and simultaneously, establishing the statute of female haemophilia worldwide [7,8].

The prevalence of haemophilia in the world is a very heterogeneous domain. In the global survey (2021) of the World Federation of Haemophilia (WFH), 117 National Haemophilia Associations reported a total number of 256,840 PwH, 185,318 with HA (3.39% being females), and 37,998 with HB (5.53% being females) [9]. A more comprehensive epidemiological survey, conducted using national registries in Australia, Canada, France, Italy, and New Zealand, focused on determining both the overall prevalence and prevalence at birth of people with hemophilia, revealed significantly higher number of patients (see Table 2) [10].

Large variability in haemophilia prevalence across countries is also dependent on race, ethnicity, socioeconomic level, and quality of health care. Taking into account the life expectancy of people with hemophilia (PwH), which is notably lower by approximately 64%, 57.7%, and 93% in countries with upper-middle, lower-middle, and low incomes, respectively, as per World Bank data, lends support to the acceptability of the overall residual prevalence estimates for hemophilia [11,12].

This entire situation is simultaneously impacting the epidemiology of female haemophilia. The long-lasting misconception that women can only be a carrier and that their level of factor coagulation assessed at 50–60% is sufficient for protection against bleeding are responsible both for the failure to recognize real haemophilia in females and for the lack of awareness regarding this diagnosis [13,14]. The carriers, even with major bleeding, are under-recognized and overlooked, and are considered simply as persons transmitting the disease, not candidates for specific therapy [15]. Since the improvement in laboratory performances and, above all, since the bio-molecular determination of the structures of the genes F8 and F9, the perception regarding diagnosis has significantly changed. In 2022, the Scientific and Standardization Committee (SSC) of the International Society on Thrombosis and Haemostasis (ISTH) agreed on the internationally standardized terminology and nomenclature for the categorization of these patients. It was clearly established, that females with coagulation factor levels < 40% are females with haemophilia and are classified as having mild (5–40%), moderate (1–5%), and severe (<1%) forms of the disease, whereas those with coagulation factor levels > 40% are classified as being symptomatic or asymptomatic carriers [16].

Regarding the number of females with haemophilia, the reported data are very different. The United Kingdom Haemophilia Center Doctors‘ Organization (UKHCD) registered 184 haemophilia A carriers and 1074 females with FVIII deficiency [17]. Casper and Lin reviewed 731 pedigrees of families with haemophilia, and for every 100 men with haemophilia, they recorded 150 somatic carriers [18]. Winikoff suggested that 10% of carriers are affected by coagulation factor levels in the haemophilia range [19]. Miller specified that only approximately 250 women and girls with haemophilia are recorded worldwide [20]. Recently published data are not only showing an increasing number of haemophilic females, but they are also revealing, using comprehensive genetic analysis, the real molecular substrate of the disease [21,22,23,24,25]. The genetic heterogeneity of the substrate of women and girls with haemophilia is reflected in the classification of their genetic explanation: homozygosity, compound heterozygosity, hemizygosity, heterozygosity with preferential X chromosome inactivation, and heterozygosity with skewed X chromosome inactivation [22,26,27,28,29,30,31,32]. The molecular exploration is also assessing new F8 or F9 mutations, clarifying challenging cases, such as haemophilia with Turner syndrome or mosaic Turner syndrome, gonadal dysgenesis, or androgen insensitivity syndrome, etc. [30,33,34,35,36,37,38,39,40]. Such confrontation with a dual diagnosis of haemophilia and Turner syndrome in women and girls is exceptionally rare. With the advent of WES, the acceptance of this challenging association has been increased. However, in real life, the number of reported cases is very low. An extensive literature review of 147 patients, adding 5 from the author’s personal experience with Turner syndrome associated with a second genetic condition, revealed a large variety of comorbidities. Interestingly, 24% of these comorbidities were connected to X-linked diseases, yet none were associated with hemophilia. In the literature, there are only about 8 clinical cases including haemophilia associated with Turner syndrome [41].

We have presented a challenging patient, considered a heterozygous case with a lack of XCI; one X chromosome has a missense mutation of gene F8; the other chromosome X has a structural abnormality with an isochromosome X-i(Xq), with 2 long arms. The gene X inactive specific transcript (XIST), positioned on the long arm of the isochromosome, is interfering with the lyonization, which is a normal process of XCI; consequently, the X chromosome with the haemophilic mutation remained active, expressing clinically severe haemophilia. Unexpectedly, the structural chromosomal abnormality was at the same time responsible for Turner syndrome, defined in 45% of cases by the karyotype 45,X0; i(Xq) was reported in 15–18% of cases, the other structural defects (ring chromosome 46,X,r(X), deletion 46,X,(Xp or Xq), mosaicism (45,X/46,XX, 45,X/46,XY or 45,X/47,XXX) being much rarer. The development of gonadal dysfunction and autoimmune complications (thyroiditis, Graves’ disease, juvenile rheumatoid arthritis, hearing decline) have been observed in such cases in later age. Our patient has only a significant stature deficiency. However, her continuous monitoring would be able to reveal other potential aspects in the future.

It is noteworthy to mention the disparity between the phenotype of haemophilia of father and daughter. The father, who has moderate severity disease, shows frequent muscular-cutaneous and joint bleedings. The daughter is constantly with FVIII < 1% without inhibitors, but she is not experiencing significant bleeding events. That was probably the reason why, despite her condition of an obligate carrier, she was not checked for FVIII status, and the discovery of severe haemophilia happened only after the age of 8 years.

A coincidental occurrence of severe haemophilia A and Turner syndrome is extremely rare, with the PubMed database containing not more than 10 reported cases (Table 3) [37,38,39,40,41,42].

The characteristic of our case is the occurrence of the structural chromosomal abnormality, i(Xq), responsible for the development of the Turner syndrome, and at the same time, for a detrimental transformation of an obligate carrier into a severe haemophilia, two distinct provocative pathological entities. What makes our patient a particular case is their challenging condition. She does not present a significant clinical expression. Consequently, she can remain undiagnosed and can be confronted with life-threatening bleeding events which could be uncontrolled without a specific replacement therapy for haemophilia. Moreover, along the years connected to her Turner syndrome, she can develop a lot of disabilities (learning difficulties, cardiac abnormalities, gonadal or renal failure, autoimmune comorbidities, etc.), all of them preventable with proper therapy.

## 4. Conclusions

It is of decisive importance to have a correct and timely diagnosis of female haemophilia, above all for their potentially life-threatening bleedings during surgical interventions, accidents, and major menstrual or postpartum bleeding [42,43,44]. In female haemophilia, the approach has to be the same as in male PwH, requiring the same treatment, often with life-saving prophylactic replacement with the missing factor. Therefore, the leading way forward can be achieved by raising awareness for female haemophilia, through diagnostic identification, which is possible by increasing haemostatic exploration and genetic screening supporting post-diagnosis with pre-conceptional counseling, as well as proper monitoring of pregnancy, delivery, and postpartum care. Mandatory attention should be dedicated to all potential or obligatory carriers belonging to families with a history of haemophilia. In this frame, a molecular genetic analysis represents an essential tool in elucidating the substrate of the heterogeneous diverse mechanisms of a female phenotype, which is important for the prediction of patients’ evolution and of her potential children.

## Figures and Tables

**Table 1:** Successive diagnosis of the patient.

Initial Lab Results	Current Investigations	Biomolecular Investigations	Cytogenetics
- aPTT 52 s, prothrombin time-14.4 s, IP 88.2%, INR-1.01; - repeated bruises and hematomas since childhood/prolonged post-dental extraction bleeding	- aPTT-47.5 s, prothrombin time-13.3 s, thrombin time-13.1 s, FVIII <1%, FIX-57.4%, factor von Willebrand activity-48.4%, von Willebrand antigen-46.5%, ristocetin cofactor-75%	missense mutation of F8 within the last base of exon 14, at codon 391—c1172G>A p.R.391H heterozygous	Isochromosome X-46,X,i(Xq)
Asymptomatic carrier/symptomatic carrier	Severe haemophilia A	Confirmed severe haemophilia A	Turner syndrome

**Table 2:** Data regarding the general prevalence and prevalence at birth of haemophilia.

	Haemophilia A	Haemophilia B
General prevalence/100,000 males	17.1(14.8–19.3)	3.8 (3.2–4.4)
General prevalence at birth/100,000 males	23.2(20.1–26.3)	4.7 (3.4–6.1)
Correction for underestimation of diagnosed cases in a 5-year lag time	24.6 (21.4–27.7)	5.0 (3.6–6.5)

**Table 3:** Synopsis of reported cases with concomitant Haemophilia A and Turner Syndrome.

No	Age	Sex	Family History	Patient Diagnosis	Genetic Defect	Publication
1	6 months	female	negative	Mosaic Turner syndrome (45 XO) with ring X (p22, 2q13) with severe haemophilia A and persistent hyperplastic primary vitreous.	45 XO with ring X chromosome 46X: rX (p22, 2q13); intron-22-inversion (F8, IVS22 INV) hemizygote	Shahriari, 2016 [38]
2	2 years	female	negative	Mild haemophilia A and Turner’s syndrome	F8 missense mutation c.5123G>A (p.Arg1708His) in exon 14; hemizygosity for the X-chromosome	Williams, 2012 [38]
3	3 months	female	carrier mother	Severe Haemophilia A and Turner Syndrome	intron-22-inversion (F8, IVS22 INV); 45,X0 karyotype	Weinspach, 2009 [37]
4	3 months	female	negative	Turner syndrome and moderate haemophilia A	46,X,idic(X)(p11) karyotype; F8 de novo mutation	Panarello, 1992 [39]
5	7 months	female	carrier mother	Severe haemophilia A and Turner’s syndrome	intron-22-inversion (F8, IVS22 INV); 45,X0 karyotype	Sasanakul, 1999 [40]
6	NA	female	NA	Severe haemophilia A in a phenotypically normal female with 45,X/46,Xr(X) mosaicism	45,X/46,Xr(X) mosaicism karyotype	Ariyoshi, 1985 [38]
7	5 years	female	carrier mother, brother with severe haemophilia A	Severe haemophilia A and Turner’s syndrome	deletion in Xq28 region; 45,X/46,XXr mosaicism karyotype	Gilgenkrantz, 1986 [38]
8	6 years	female	carrier mother	Haemophilia A in a phenotypically normal female with XX-XO mosaicism	45,X/46,XX mosaicism karyotype	Gilchrist, 1965 [38]
9	preterm, gestational age 28 weeks	female	mother’s brother had severe haemophilia	Severe haemophilia A in a preterm girl with Turner syndrome	mosaic karyotype (46,X + mar,45, X)	Berendt, 2020 [38]

## Data Availability

Publicly available datasets were analyzed in this study.

## References

[1] Tosetto A., Castaman G., Rodeghiero F. (2013). Bleeders, bleeding rates, and bleeding score. J. Thromb. Haemost..

[2] Weyand A.C., Sidonio R.F., Sholzberg M. (2022). Health issues in women and girls affected by haemophilia with a focus on nomenclature, heavy menstrual bleeding, and musculoskeletal issues. Haemophilia.

[3] d’Oiron R., O’Brien S., James A.H. (2021). Women and girls with haemophilia: Lessons learned. Haemophilia.

[4] Khair K., Holland M., Pollard D. (2013). The experience of girls and young women with inherited bleeding disorders. Haemophilia.

[5] Sager R. (2014). Women with haemophilia: More than just carriers. J. Haemoph. Pract..

[6] Presky K.O., Kadir R.A. (2020). Women with inherited bleeding disorders—Challenges and strategies for improved care. Thromb. Res..

[7] Blanchette V.S., Srivastava A. (2015). Definitions in hemophilia: Resolved and unresolved issues. Semin. Thromb. Hemost..

[8] Stonebraker J.S., Bolton-Maggs P.H., Soucie J.M., Walker I., Brooker M. (2010). A study of variations in the reported haemophilia A prevalence around the world. Haemophilia.

[9] https://wfh.org/research-and-data-collection/annual-global-survey/.

[10] Iorio A., Stonebraker J.S., Chambost H., Makris M., Coffin D., Herr C., Germini F., Data and Demographics Committee of the World Federation of Hemophilia (2019). Establishing the Prevalence and Prevalence at Birth of Hemophilia in Males: A Meta-analytic Approach Using National Registries. Ann. Intern. Med..

[11] Soucie J.M., Miller C.H., Dupervil B., Le B., Buckner T.W. (2020). Occurrence rates of haemophilia among males in the United States based on surveillance conducted in specialized haemophilia treatment centres. Haemophilia.

[12] Inserro A. Prevalence of Hemophilia Worldwide is Triple That of Previous Estimates. New Study Says, AJMC, 2019, Life Sciencies. https://www.ajmc.com/view/prevalence-of-hemophilia-worldwide-is-triple-that-of-previous-estimates-new-study-says.

[13] Soucie J.M., Evatt B., Jackson D., The Hemophilia Surveillance System Project Investigators (1998). Occurrence of hemophilia in the United States. Am. J. Hematol..

[14] Raso S., Lambert C., Boban A., Napolitano M., Siragusa S., Hermans C. (2020). Can we compare haemophilia carriers with clotting factor deficiency to male patients with mild haemophilia?. Haemophilia.

[15] Blanchette V.S., Key N.S., Ljung L.R., Manco-Johnson M.J., van den Berg H.M., Srivastava A. (2014). Definitions in hemophilia: Communication from the SSC of the ISTH. J. Thromb. Haemost..

[16] van Galen K.P.M., d’Oiron R., James P., Abdul-Kadir R., Kouides P.A., Kulkarni R., Mahlangu J.N., Othman M., Peyvandi F., Rotellini D. (2021). A new hemophilia carrier nomenclature to define hemophilia in women and girls: Communication from the SSC of the ISTH. J. Thromb. Haemost..

[17] (2019). Annual Report, Women with Haemophilia Also Exist. http://www.ukhcdo.org.

[18] Kasper C.K., Lin J.C. (2010). How many carriers are there?. Haemophilia.

[19] Winikoff R., Amesse C., James A., Lee C., Pollard D. (2004). The role of haemophilia treatment centres in providing services to women with bleeding disorders. Hemophilia.

[20] Seeler R.A., Vnencak-Jones C.L., Bassett L.M., Gilbert J.B., Michaelis R.C. (1999). Severe haemophilia A in a female: A compound heterozygote with nonrandom X-inactivation. Haemophilia.

[21] Miller C.H., Bean C.J. (2021). Genetic causes of haemophilia in women and girls. Haemophilia.

[22] Miller C.H., Soucie J.M., Byams V.R., Payne A.B., Sidonio R.F., Buckner T.W., Bean C.J. (2021). Women and girls with haemophilia receiving care at specialized haemophilia treatment centres in the United States. Haemophilia.

[23] Pavlova A., Brondke H., Müsebeck J., Pollmann H., Srivastava A., Oldenburg J. (2009). Molecular mechanisms underlying hemophilia A phenotype in seven females. J. Thromb. Haemost..

[24] Pezeshkpoor B., Oldenburg J., Pavlova A. (2022). Insights into the Molecular Genetic of Hemophilia A and Hemophilia B: The Relevance of Genetic Testing in Routine Clinical Practice. Hamostaseologie.

[25] Janczar S., Babol-Pokora K., Jatczak-Pawlik I., Taha J., Klukowska A., Laguna P., Windyga J., Odnoczko E., Zdziarska J., Iwaniec T. (2020). Six molecular patterns leading to hemophilia A phenotype in 18 females from Poland. Thromb. Res..

[26] Janczar S., Kosinska J., Ploski R., Pastorczak A., Wegner O., Zalewska-Szewczyk B., Paige A.J., Borowiec M., Mlynarski W. (2016). Haemophilia A and cardiovascular morbidity in a female SHAM syndrome carrier due to skewed X chromosome inactivation. Eur. J. Med. Genet..

[27] Radic C.P., Rossetti L.C., Abelleyro M.M., Tetzlaff T., Candela M., Neme D., Sciuccati G., Bonduel M., Medina-Acosta E., Larripa I.B. (2015). Phenotype-genotype correlations in hemophilia A carriers are consistent with the binary role of the phase between F8 and X-chromosome inactivation. J. Thromb. Haemost..

[28] Coleman R., Genet S.A., Harper J.I., Wilkie A.O. (1993). Interaction of incontinentia pigmenti and factor VIII mutations in a female with biased X inactivation, resulting in haemophilia. J. Med. Genet..

[29] Martín-Salces M., Venceslá A., Alvárez-Román M.T., Rivas I., Fernandez I., Butta N., Baena M., Fuentes-Prior P., Tizzano E.F., Jiménez-Yuste V. (2010). Clinical and genetic findings in five female patients with haemophilia A: Identification of a novel missense mutation, p.Phe2127Ser. Thromb. Haemost..

[30] Knobe K.E., Sjörin E., Soller M.J., Liljebjörn H., Ljung R.C. (2008). Female haemophilia A caused by skewed X inactivation. Haemophilia.

[31] Ljung R.C., Sjörin E. (1999). Origin of mutation in sporadic cases of haemophilia A. Br. J. Haematol..

[32] Zheng J., Ma W., Xie B., Zhu M., Zhang C., Li J., Wang Y., Wang M., Jin Y. (2015). Severe female hemophilia A patient caused by a nonsense mutation (p. Gln1686X) of F8 gene combined with skewed X-chromosome inactivation. Blood Coagul. Fibrinolysis.

[33] Afrose S. (2017). Haemophilia A in a female patient with Turner syndrome. Haematol. J. Bangladesh.

[34] Chan J.T., Cabanas M.C.C. (2021). A Rare Variant of Turner Syndrome with Isodicentric X Chromosome Resulting in Trisomy: A Case Report. J. Endocr. Soc..

[35] Cui X., Cui Y., Shi L., Luan J., Zhou X., Han J. (2018). A basic understanding of Turner syndrome: Incidence, complications, diagnosis, and treatment. Intractable Rare Dis. Res..

[36] Gursoy S., Ercal D. (2017). Turner syndrome and its variants. J. Pediatr. Res..

[37] Weinspach S., Siepermann M., Schaper J., Sarikaya-Seiwert S., Rieder H., Gerigk M., Höhn T., Laws H.J. (2009). Intracranial hemorrhage in a female leading to the diagnosis of severe hemophilia A and Turner syndrome. Klin. Padiatr..

[38] Berendt A., Wójtowicz-Marzec M., Wysokińska B., Kwaśniewska A. (2021). Severe haemophilia A in a preterm girl with Turner syndrome: Case report—A diagnostic and therapeutic challenge for a paediatrician (Part 2). Ital. J. Pediatr..

[39] Panarello C., Acquila M., Caprino D., Gimelli G., Pecorara M., Mori P.G. (1992). Concomitant Turner syndrome and hemophilia A in a female with an idic(X)(p11) heterozygous at locus DXS52. Cytogenet. Cell Genet..

[40] Chuansumrit A., Sasanakul W., Goodeve A., Treratvirapong T., Parinayok R., Pintadit P., Hathirat P. (1999). Inversion of intron 22 of the factor VIII gene in a girl with severe hemophilia A and Turner’s syndrome. Thromb. Haemost..

[41] Jones K.L., McNamara E.A., Longoni M., Miller D.E., Rohanizadegan M., Newman L.A., Hayes F., Levitsky L.L., Herrington B.L., Lin A.E. (2018). Dual diagnoses in 152 patients with Turner syndrome: Knowledge of the second condition may lead to modification of treatment and/or surveillance. Am. J. Med. Genet. Part A.

[42] Byams V.R., Kouides P.A., Kulkarni R., Baker J.R., Brown D.L., Gill J.C., Grant A.M., James A.H., Konkle B.A., Maahs J. (2011). Surveillance of female patients with inherited bleeding disorders in United States Haemophilia Treatment Centres. Haemophilia.

[43] Chi C., Lee C.A., Shiltagh N., Khan A., Pollard D., Kadir R.A. (2008). Pregnancy in carriers of haemophilia. Haemophilia.

[44] McLintock C. (2018). Women with bleeding disorders: Clinical and psychological issues. Haemophilia.

